# PRELP secreted from mural cells protects the function of blood brain barrier through regulation of endothelial cell-cell integrity

**DOI:** 10.3389/fcell.2023.1147625

**Published:** 2023-10-23

**Authors:** Hongorzul Davaapil, Jack Hopkins, Nadia Bonnin, Vasiliki Papadaki, Alex Leung, Hirofumi Kosuge, Takumi Tashima, Makoto Nakakido, Ryohei Sekido, Kouhei Tsumoto, Mandeep S. Sagoo, Shin-Ichi Ohnuma

**Affiliations:** ^1^ UCL Institute of Ophthalmology, UCL, London, Untited Kingdom; ^2^ Institute of Medical Science, The University of Tokyo, Tokyo, Japan; ^3^ NIHR Biomedical Research Centre for Ophthalmology, Moorfields Eye Hospital, London, Untited Kingdom; ^4^ Retinoblastoma Genetic Screening Unit, Barts Health NHS Trust, Royal London Hospital, London, Untited Kingdom

**Keywords:** PRELP, vasculature, neuroinflammation, integrity, adhesion, EndMT, BBB

## Abstract

**Introduction:** Proline/arginine-rich end leucine-rich repeat protein (PRELP), is a small secreted proteoglycan expressed by pericytes and vascular smooth muscle cells surrounding the brain vasculature of adult mouse.

**Methods:** We utilised a *Prelp* knockout (*Prelp* ^−/−^) mouse model to interrogate vasculature integrity in the brain alongside performing in vitro assays to characterise PRELP application to endothelial cells lines. Our findings were supplemented with RNA expression profiling to elucidate the mechanism of how PRELP maintains neurovasculature function.

**Results:** *Prelp* ^−/−^ mice presented with neuroinflammation and reducedneurovasculature integrity, resulting in IgG and dextran leakage in the cerebellum and cortex. Histological analysis of *Prelp* ^−/−^ mice revealed reducedcell-cell integrity of the blood brain barrier, capillary attachment of pericytes andastrocyte end-feet. RNA-sequencing analysis found that cell-cell adhesion andinflammation are affected in *Prelp* ^−/−^ mice and gene ontology analysis as well as gene set enrichment analysis demonstrated that inflammation related processes and adhesion related processes such as epithelial-mesenchymal transition and apical junctions were significantly affected, suggesting PRELP is a regulator of cell-cell adhesion. Immunofluorescence analysis showed that adhesion junction protein expression levels of cadherin, claudin-5, and ZO-1, was suppressed in *Prelp* ^−/−^ mice neurovasculature. Additionally, *in vitro* studies revealed that PRELP application to endothelial cells enhances cell-cell integrity, induces mesenchymal-endothelial transition and inhibits TGF-β mediated damage to cell-cell adhesion.

**Discussion:** Our study indicates that PRELP is a novel endogenous secreted regulator of neurovasculature integrity and that PRELP application may be a potential treatment for diseases associated with neurovascular damage.

## 1 Introduction

The blood-brain barrier (BBB) is a tight functional barrier composed of capillary endothelial cells, astrocytes, pericytes, and neurons, which prevents neurotoxic plasma components, blood cells, and pathogens from entering the brain (Weiss et al., 2009; Sweeney et al., 2019). Defects in the integrity of the BBB results in the accumulation of toxic molecules leaked from the vasculature in the brain. This leakage causes central nervous system (CNS) diseases such as Alzheimer’s disease, Huntington’s disease, and stroke (Weiss et al., 2009; Sweeney et al., 2019). The BBB is characterized by strong cell-cell adhesions through adherens and tight junctions (Alahmari, 2021). These dynamic structures are governed by diverse proteins secreted from the neuro vascular components, which trigger downstream signalling events involved in cytoskeletal reorganization, and endothelial permeability (Gaengel et al., 2009). The endothelial-to-mesenchymal transition (EndMT) has been recognized as a major biological event that controls vascular leakage (Cho et al., 2018; Man et al., 2019; Piera-Velazquez and Jimenez, 2019).

Recently, we have reported that two secreted proteoglycans, proline/arginine-rich end and leucine-rich protein (PRELP) and osteomodulin (OMD) function as inhibitors of bladder cancer initiation by inhibiting epithelial-mesenchymal transition (EMT) and by activating cell-cell adhesion of bladder epithelial cells (Papadaki et al., 2020). Intriguingly, *PRELP* expression was regulated by epigenetically through acetylation of lysine residue 5 of histone H2B in the *PRELP* gene promoter region in bladder cancer (Shozu et al., 2022). In addition, we demonstrated that Prelp was expressed in mouse retina and loss of Prelp contributed to retinoblastoma cell progression by reducing cell-cell adhesion and facilitated EMT (Hopkins et al., 2022). We further investigated the roles in other tumors and identified that PRELP showed a tumor suppressive role by regulated PI3K-AKT signalling pathway in high-grade ovarian cancer (Dozen et al., 2022).

PRELP is a class II member of the small leucine rich proteoglycan (SLRP) family (Dellett et al., 2012; Iozzo and Schaefer, 2015). SLRP family members bind various extracellular proteins such as TGF-β, BMP, EGF, IGF, Wnt, and collagens and can regulate multiple signalling pathways in context dependent manners (Morris et al., 2007; Dellett et al., 2012; Chacon-Solano et al., 2022; Lopez and Bonassar, 2022). They are involved in various biological processes such as cancer, inflammation, and development (Birke et al., 2014; Luehders et al., 2015; Papadaki et al., 2020). In this paper, we report that PRELP is selectively expressed in mural cells around the neurovasculature and contributes to the regulation of BBB integrity. PRELP depletion in mouse brain caused blood leakage, indicating that PRELP is responsible for BBB integrity. Our results suggest that PRELP could be used as a new strategy to inhibit neurovascular leakage or protect BBB dysfunction against neurological disorders.

## 2 Materials and methods

For Antibodies, reagents, data accession number, software, and primes, all antibodies, reagents, accession number, software and algorithms, and primes used in this study were listed in Supplementary Tables S1, S2.

### 2.1 *Omd* ^−/−^ and *Prelp* ^−/−^ mice

Mouse lines were generated by Takeda Pharmaceutical Company and wild type and heterozygote founders were imported to our animal facility. All animal procedures were performed in accordance to the Animals (Scientific procedures) Act 1986 of the United Kingdom Government and housed in compliance with the Home Office Code of Practice. Mice were kept in individually ventilated cages (IVCs), in a 12 h light: dark cycle and were fed a complete pelleted mouse diet and with constant access to water.

Briefly, *Omd* ^*flox*^ or *Prelp* ^*flox*^ ES cells were generated from C57BL/6J ES cells by homologous recombination. Targeting vectors were constructed by insertion of the first LoxP sequence upstream of exon two, containing the initiation codon on the *Omd* or *Prelp* locus. A second LoxP sequence, neomycin resistant unit, and LacZ unit was inserted downstream of exon three. Cre expression plasmid was electroporated into the recombinant flox ES cells to generate ES cells harboring the knockout allele. The resulting cells were injected into ICR tetraploid blastocysts to generate chimeric male mice which were backcrossed to C57BL/6J females. Single knockout mice (*Omd* ^*LacZ/LacZ*^ and *Prelp* ^*LacZ/LacZ*^) were generated by cross breeding within the colony.

Genotyping: Genotyping PCR reactions were performed as follows. Mouse ear punches were mixed with 180 μL DirectPCR Lysis Reagent (Viagen Biotech) and 0.4 mg/mL Proteinase K (Sigma) solution before rocking at 55°C overnight. The samples were then incubated at 85°C for 45 min before centrifugation and collection of the resulting lysate. OMD samples were genotyped using an Invitrogen kit. A master mix was prepared according to the manufacturers protocol using the primers described in Supplementary Table S2 and added to 5 μL genomic DNA. Samples were incubated at 95°C for 3 min prior to 35 cycles consisting of 30 s at 95°C, 90 s at 61°C and 90 s at 72°C. The final extension was 10 min at 72°C. This produced amplicons of different lengths: 298 bp in wild type mice with OMD-A and OMD-B2 primers; 541 bp in knockout mice with OMD-B2 and LacZ-5756 primers; or both amplicons present in heterozygotic mice. PRELP samples were genotyped using a Multiplex PCR kit (Qiagen). A master mix was prepared according to the manufacturers protocol using the primers described in Supplementary Table S2 and added to 2.5 μL genomic DNA. Samples were incubated at 95°C for 15 min prior to 40 cycles consisting of 30 s at 94°C, 90 s at 63°C and 90 s at 72°C. The final extension was 10 min at 72°C. Amplicons were 846 bp for wild type mice with PRELP-A and PRELP-B primers, 634 bp for knockout mice with PRELP-C and LacZ-B primers, or both amplicons present in heterozygotic mice.

### 2.2 Tissue processing and staining

Mouse brains were isolated and fixed in 4% PFA for 24 h before paraffin embedding and sectioning. Tissue for paraffin sectioning was processed in the Institute’s Pathology department using an automated machine (Leica ASP300S). Samples were sectioned into 5 μm slices on superfrost slides treated with poly-L-lysine, dried and stored at room temperature. Histological staining was performed in an automated system in the Pathology department. H&E, von Kossa, alcian blue, congo red and MSB, were performed in the Pathology department following department’s specific protocol for each stain. Methylene blue and basic fuchsin staining was performed on semi-thin sections of xenografted tumors. For immunostaining, slides were dewaxed for 10 min in Histoclear and rehydrated in an ethanol-water graded series. Antigen retrieval was performed by boiling the samples for 15 min in citrate (pH 6.0) or Tris-EDTA buffer (pH 9.0) depending on the antibody. Sections were blocked for 1 h in 10% goat serum in PBS and were incubated overnight with primary antibodies at 4°C. Detection was performed by incubation with anti-rabbit or anti-mouse Alexa Fluor 488 secondary antibodies, for 1 h at room temperature (1:500 dilution, Life technologies).

### 2.3 β-Galactosidase analysis

Mouse brains were isolated from adult mice and were fixed in 4% PFA at 4°C briefly for 2 h with gentle agitation. Afterwards they were washed in PBS and left at 30% sucrose at 4°C overnight before subsequently frozen in OCT. Cryosections 10 μm thick were washed twice in PBS +2 mM MgCl_2_ for 20 min and were stained overnight in X-gal at 37°C. Sections were washed in PBS and then either counterstained and mounted in Nuclear Fast Red (Vector Laboratories) or followed by IHC. For IHC, briefly, samples were immediately blocked for 1 h with 10% goat serum and were incubated with the primary antibodies overnight. Secondary staining was completed with anti-rabbit or anti mouse Alexa Fluo-488 antibodies, counterstained in DAPI and mounted.

### 2.4 Immunocytochemisty

Coverslips with cell monolayers were washed in PBS and fixed with either 4% PFA or ice-cold methanol for 10 min. Samples were then washed and incubated for 1 h in blocking buffer. After blocking and incubation with primary antibodies overnight at 4°C, the samples were incubated with secondary Alexa Fluor 488 (1:500, Life Technologies) in blocking buffer for 1 h at room temperature, counterstained with Hoechst solution (Invitrogen) and mounted. Slides were imaged using a Zeiss LSM710 at ×10 and ×40 magnification. After laser intensity settings were optimized, images were processed on ImageJ and a standard threshold to remove background noise across all samples.

### 2.5 Microscope settings and image analysis

A preliminary analysis determined the thickness of samples, and the microscopic settings were adjusted accordingly to enable the detection of structures. In this setting, Hamamatsu ORCA-ER Digital Camera (Hamamatsu, Japan) and μManager software (Edelstein et al., 2010) were used to obtain fluorescent images on an Axioskop 2 Plus (Zeiss, Germany) from sections. A ×40 differential interference contrast objective with an aperture of 0.95 and 0.25 working distance and 10X ocular lens were used to obtain each region of interest. Imaging parameters of laser intensity and exposure time were optimized and uniformly set in the same experiments. Images were then processed, despeckled, background subtracted and applied with a median filter at 2px in order to remove all background noise. Huang auto threshold was then applied, and the threshold was saved as an ROI. This ROI was then used as the “outline” of immunostaining to measure, and regions of interest measured and quantified with FIJI software.

### 2.6 Vascular capillaries leakage studies

25 mg/mL 70 kDa Dextran-Texas Red was injected into mice by intravenously. After circulation for 3 h, mice were culled and brain tissues harvested. Perfusion was performed through the heart before collecting tissues. Dye excess was washed out through fixation and washing in PBS before cryoprotection. Sample preparation was done as described above. Vascular permeability was then visualized with fluorescence microscopy. Vessels were traced on ImageJ and interior staining removed. The resulting external staining was then quantified by methods mentioned above.

### 2.7 Expression profiling of meningeal vessels in RNA-seq analysis

#### 2.7.1 Sample preparation

Four wild-type mice, three *Omd* ^−/−^ and three *Prelp* ^−/−^ knockout mice meningeal samples were used for RNA-seq analysis. RNA was extracted via ARCTURUS PicoPure RNA Isolation kit for mouse samples or PureLinkTM RNA Mini Kit for HUVECs. In brief, meningeal vessels were excised and homogenized using a rotor-stator homogenizer. After centrifugation at 3,000 × g for 2 min and extracted in accordance with manufacturer’s instructions RNA was quantified and qualified by Agilent’s 2200 TapeStation, measuring RNA concentration and agarose gel electrophoresis.

#### 2.7.2 Library preparation

Samples were processed using the KAPA mRNA HyperPrep Kit (Roche KK8580) according to manufacturer’s instructions.

Briefly, mRNA was isolated from total RNA using Oligo dT beads to pull down poly-adenylated transcripts. The purified mRNA was fragmented using chemical hydrolysis (heat and divalent metal cation) and primed with random hexamers. Strand-specific first strand cDNA was generated using Reverse Transcriptase in the presence of Actinomycin D. This allows for RNA dependent synthesis while preventing spurious DNA-dependent synthesis. The second cDNA strand was synthesized using dUTP in place of dTTP, to mark the second strand. The resultant cDNA is then “A-tailed” at the 3’ end to prevent self-ligation and adapter dimerization. Full length xGen adaptors (IDT), containing two unique 8 bp sample specific indexes, a unique molecular identifier (N8) and a T overhang are ligated to the A-Tailed cDNA. Successfully ligated cDNA molecules were then enriched with limited cycle PCR (50 ng of starting material, 15 PCR cycles). The high fidelity polymerase employed in the PCR is unable to extend through uracil. This means only the first strand cDNA is amplified for sequencing, making the library strand specific (first-strand).

#### 2.7.3 Sequencing

Libraries to be multiplexed in the same run are pooled in equimolar quantities, calculated from Qubit and Bioanalyser fragment analysis.

Samples were sequenced on the NextSeq 500 instrument (Illumina, San Diego, US) using a 75 bp single read run with a corresponding 16 bp UMI read.

#### 2.7.4 Data analysis

Run data were demultiplexed and converted to fastq files using Illumina’s bcl2fastq Conversion Software v2.19. Fastq files were then aligned to the *Mus musculus* genome GRCm38 or *Homo sapiens* genome GRCh38 using RNA-STAR 2.5.2b then UMI deduplicated using Je-suite (1.2.1). Reads per transcript were counted using FeatureCounts and differential expression was estimated using Galaxy. Log2 fold change and *p* values of pairwise differential expression between wild type samples and knockout samples or PRELP treated and untreated samples were then analysed using Qiagen’s Ingenuity Pathway Analysis (version 48207413).

### 2.8 Measurement of microglial morphological change

Microglial morphological change was measured using the previously reported protocol with minor modifications (Morrison and Filosa, 2013). Briefly, images of Iba-1 staining were processed to remove noise and any background staining. A threshold was applied to produce a binary image and skeletonized which was analysed using Analyse Skeleton plugin. The image was then analysed, and the sum of all branch lengths were extracted and used for further quantification. All images were processed using the same parameters. Branch lengths were normalized by number of microglia per area and the resulting value was expressed as branch length per microglial density.

### 2.9 Brain integrity studies

Transepithelial/transendothelial electrical resistance (TEER) were used to assess the brain integrity using an EVOM Volt-Ohmeter (World Precision Instruments). STX2 probes were disinfected and air-dried before being inserted into upper and bottom chambers. Resistance was measured for empty wells containing media before determined across monolayers. Values were calculated by multiplying raw values by transwell growth area once the base value for empty wells had been subtracted. 70,000 cells per 24-well transwel insert were seed and allow them to grow for 4 days in control and PRELP conditioned media. TEER measurements were completed 96 h after PRELP treatment.

For the membrane permeability, permeability assays were completed using an *in vitro* vascular permeability assay kit (Merck). In brief, inserts were hydrated and seeded with 0.5 × 10^5^ cells/insert in media and incubated until a monolayer formed. PRELP conditioned media was applied and incubated for 24 h. FITC-Dextran was added to each insert and incubated for 20 min in the dark before the insert was removed from the well. Media in the well was mixed and transferred to black 96-well opaque plate to measure fluorescence intensity at 485 nm and 535 nm excitation and emission respectively.

### 2.10 Experimental design and statistical analysis

Experiments were performed at least in three independent replicates. All data shown as the mean ± SEM. All data was tested for normal distribution before a Student’s T-test was used to calculate significance. A two-tailed student-t test was used for statistical analysis. *p < 0.05, **p < 0.01 and ***p < 0.001. NS, not significant. Imaging data were analysed by NIH software ImageJ (Ljosa et al., 2012). N shown in the figure legends indicates the number of animals used in the experiments. For image analyses, five fields were randomly imaged per animal.

### 2.11 Data availability

The data that support the findings of this study are available from the Gene Expression Omnibus (GEO) (GSE199122) and from the corresponding author upon reasonable request.

## 3 Results

### 3.1 PRELP is selectively expressed in vascular smooth muscle cells (vSMCs) and pericytes around brain vasculature

**FIGURE 1:**
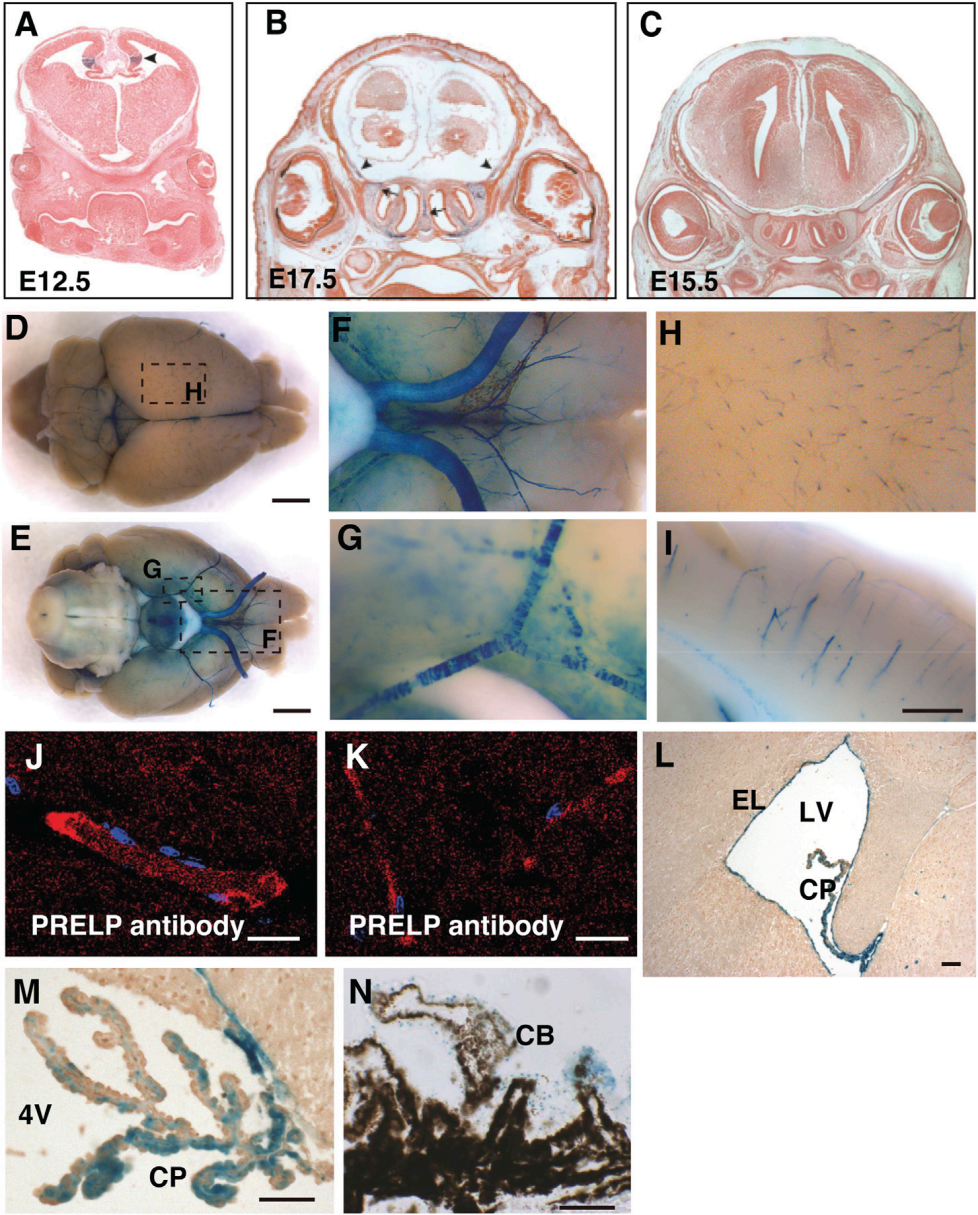
PRELP is expressed mural cells around brain vasculatures and ependymal cells in mouse brain. **(A–C)** Embryonic PRELP expression of head. Sections of *Prelp* ^*+/LacZ*^ embryos were stained for X-gal. **(A)** Embryonic day 12.5 (E12.5). Arrowhead indicate cortical hem. **(B)** Embryonic day 17.5 (E17.5). Arrow indicate the nasal septum and arrowhead indicate the sites of bone formation **(C)** Embryonic day 15.5 (E15.5). **(D–I)** Whole-mount X-gal staining of adult *Prelp* ^*+/LacZ*^ brains. **(D–E)** Dorsal and ventral views. X-gal staining is observed in the blood vessels. Scale bar: 2 mm. **(F)** Magnified from **(E)** around the ventral-posterior area. **(G)**, Magnified from **(E)** around ventral lateral. **(H)** Magnified from **(D)** around cerebrum. **(I)** Sagittal section of X-gal stained brain around cerebellum. Scale bar: 500 μm. **(J–K)** Anti−PRELP antibody staining of rat brain. Staining is visible in large arterioles/venules **(J)** and **(K)** capillaries Scale bar: 20 μm. **(L)** Section around lateral ventricle (LV). Ependymal layer (EL). Choroid plexus (CP). Scale bar: 50 μm. **(M)** Section around 4^th^ ventricle (4V). Scale bar: 50 μm. **(N)**, Section of retina around ciliary body (CB). Scale bar: 50 μm.

PRELP expression in the CNS was examined by X-gal staining of *Prelp* ^*+/LacZ*^ mouse brain. The mouse expresses the lac-Z gene under the control of endogenous PRELP transcription elements (Papadaki et al., 2020). At embryonic stages, we observed expression in the cortical hem of the hippocampal allocortex (Figure 1A, arrowheads) and at sites of bone formation around the CNS (Figure 1B). However, we did not observe any other strong PRELP expression in the head (Figure 1C).

At the adult stages, a unique X-gal staining was observed in mural cells around the CNS vessels, covering both the dorsal and ventral part of the brain, as well as around the optic nerves, the space between the two hemispheres (superior sagittal sinus) and the central canal of the spinal cord (Figures 1D–G). Enlarged images revealed X-gal staining (Figures 1H, I), reminiscent of pericytes which encircle capillaries and vascular smooth muscle cells (vSMCs) around large arterial and venous vessels (Chen et al., 2017; Vanlandewijck et al., 2018). Staining with PRELP antibodies (α-PRELP #15) identified secreted PRELP protein largely localized around large arterioles/venules (Figure 1J) and capillaries (Figure 1K). In addition to vascular mural cells, we observed strong staining at ependymal layers of the ventricle walls (Figure 1L), the choroid plexus (Figure 1M), and non-pigmented layer of ciliary body of the retina (Figure 1N).

**FIGURE 2:**
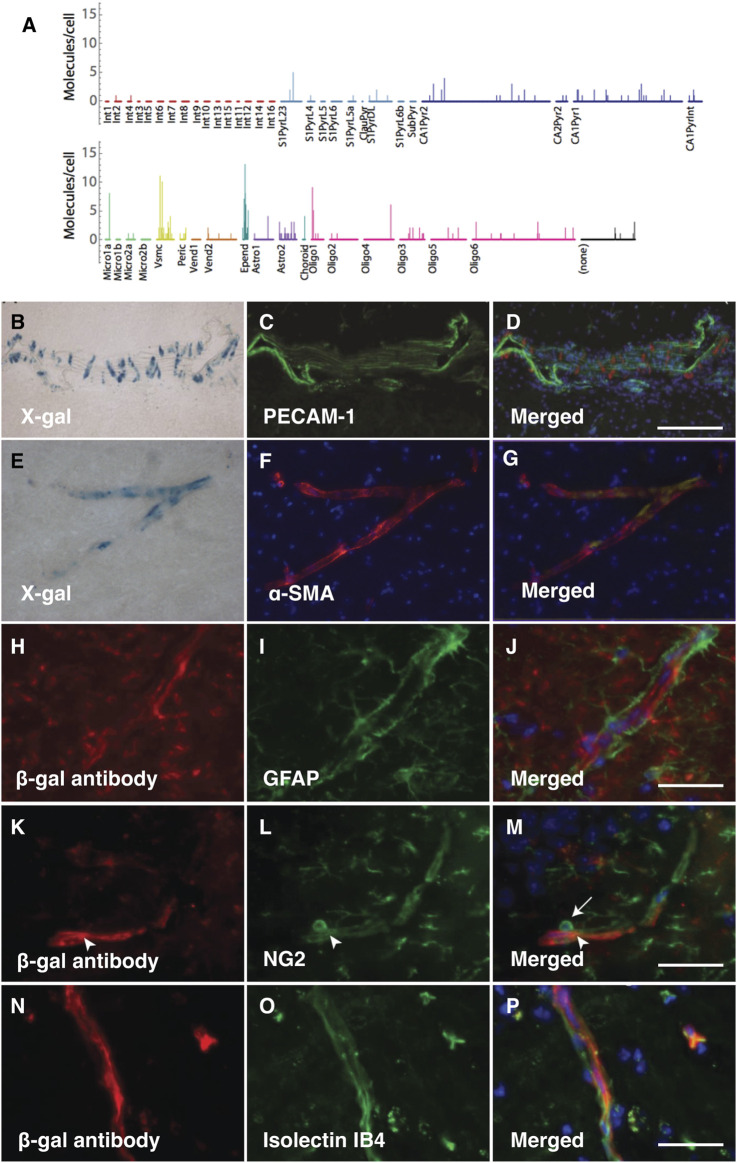
PRELP is expressed in vSMCs around vascular vessels and pericyte around capillaries. **(A)** Single cell analysis data of PRELP expression in cortical and hippocampal cells were obtained from previously published studies (Zeisel et al., 2015). Figure was generated by tool provided by the paper (Zeisel et al., 2015). **(B–D)** Double staining images with X-gal **(B)** and PECAM-1 (endothelial cell marker) antibody **(C)** of *Prelp* ^*−/+*^ large vascular. **(D)** Merged image. **(E–F)** Double staining with X-gal **(E)** and α-smooth muscle actin (α-SMA) antibody (**(F)**; vSMC marker) of *Prelp* ^−/+^ intermediate vasculature. **(G)** Merged image. **(H–J)** Double staining images with β-galactosidase (β-gal) antibody **(H)** and GFPA antibody [**(I)**; astrocyte marker) of *Prelp* ^*−/+*^ brain capillaries. **(J)** Merged image. Scale bar: 25 μm **(J,M,P)**. **(K–M)** Double staining images with β-gal antibody **(K)** and NG2 antibody **(L)**; pericyte marker) of *Prelp* ^*−/+*^ brain capillaries. **(M)** Merged image. **(N–P)** Double staining images with β-gal antibody **(N)** and Isolectin IB4 **(O)**; endothelial cell marker) of *Prelp* ^*−/+*^ brain capillaries. **(P)** Merged image. Pericyte bodies are marked by arrows. Pericyte processes are marked by arrowheads.

The analysis of *PRELP* expression in pericytes and vSMCs, but not in endothelial cells has been confirmed by published single cell mRNA expression profiling data (Zeisel et al., 2015; He et al., 2016; Vanlandewijck et al., 2018) (Figure 2A). To confirm these previously published results in this study, we performed double staining to examine where PRELP was expressed. X-gal staining did not overlap with endothelial marker PECAM-1 positive cells (Figures 2B–D). We observed that X-gal staining was co-localised with α-SMA, marking vSMCs (Figures 2E–G). There was no detectable staining of X-gal with astrocyte marker GFAP on capillaries (Figures 2H–J) although single cell analysis indicated gene expression of *PRELP* in astrocyte subpopulations (Figure 2A). Double staining with pericyte marker, NG2, showed that co-staining was limited to the pericyte processes around the vasculature (arrowhead) and not the pericyte cell body (Figure 2K–M) (arrow in Figure 2M). This suggests that the receptor gene may be expressed by pericytes—localisation with the X-gal staining (Figures 2N–P), indicating PRELP was not expressed in the endothelial cells and expressed in vSMCs and in pericytes at specific locations, rather than astrocytes surrounding vascular capillaries.

In addition to the *Prelp* ^*−/−*^ mice, we examined the vasculature in *Omd* ^*−/−*^ mice. OMD is also a class II SLRP family, highly conserved with PRELP. We previously demonstrated that OMD and PRELP are both expressed in umbrella bladder epithelial cells and involved in bladder cancer initiation in a partially redundant manner (Papadaki et al., 2020). *Omd* expression was observed across the cerebrum, but not the cerebellum, optic nerves or optic chiasm (unpublished result). *Omd* was also strongly expressed in neurons and weakly expressed in cells around vasculature (unpublished result). Thus, besides wild-type controls, we also examined *Omd* ^*−/−*^ mice as the second control.

### 3.2 Cell-cell adhesion weakened in *Prelp* ^*−/−*^ mice results in leakage from vascular capillaries in mouse brain

**FIGURE 3:**
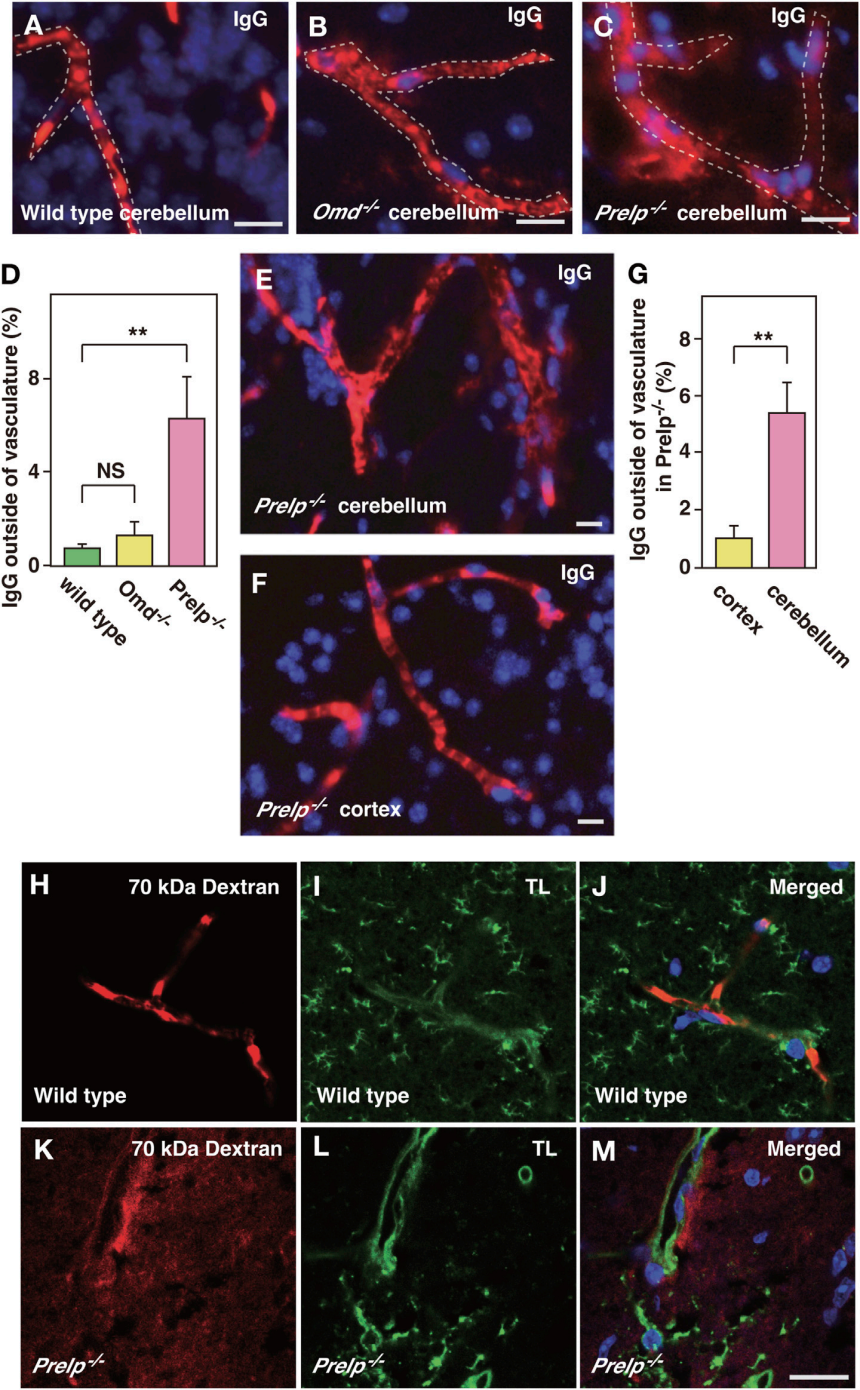
PRELP deletion results in leakage from the BBB. **(A–D)** Assessment of BBB integrity in the cerebellum by IgG staining in wild-type **(A)**, *Omd* ^−/−^ **(B)** and *Prelp* ^*−/−*^ **(C)**. Immunofluorescence was performed using anti-mouse IgG conjugated with Alexa Fluor A594 fluorophore. DAPI was used as a nuclear stain. Scale bar: 10 μm. **(D)** IgG signal outside blood vessels was quantified (*n* = 4). **(E–G)** BBB disruption in *Prelp* ^*−/−*^ is more apparent in the cerebellum. IgG and DAPI staining of *Prelp* ^*−/−*^ cerebellum **(E)** and cortex **(F)**. Scale bar: 10 μm. **(G)**, Quantified result (*n* = 3). **(H–M)** 70 kDa Dextran injection confirms BBB leakage in *Prelp* ^*−/−*^ cerebellum. Wild-type **(H–J)** and *Prekp* ^*−/−*^ **(K–M)** mice were injected with 70 kDa Dextran-Texas Red **(H,K)**. Tissues were processed and stained with tomato lectin (TL, vascular vessel and microglia marker) **(I,L)**. Merged image **(J,M)**. Scale bar: 20 μm.

vSMCs and pericytes have important roles in controlling endothelial cell-cell integrity (Armulik et al., 2010; Hayes et al., 2022), therefore, we examined the effect of PRELP deletion on leakage from neural capillaries and the BBB. Firstly, the status of vascular integrity or BBB was assessed by immunoglobulin G (IgG) staining using one-year-old mice. IgG is a 160 kDa protein, which exclusively localizes in blood plasma. The intact BBB prevents IgG from passing through the vasculature and coming into contact with neural tissues. Using anti-mouse IgG-Alexa Fluor 594, we stained for plasma IgG in wild-type, *Omd* ^*−/−*^ and *Prelp* ^*−/−*^ mouse cerebellum (Figures 3A–C). In wild type, IgG staining was retained within the vasculature and along the walls of the blood vessels in a regular striated pattern perpendicular to the length of the vessel. In the *Omd* ^*−/−*^ mice brain, diffused staining outside the vasculature was not observed (Figure 3B). However, in the *Prelp* ^*−/−*^ brain, IgG was often found to be highly diffused outside vasculature. (Figures 3C, D). This suggests that PRELP, but not OMD, is responsible for the regulation of neurovasculature integrity. Disruption of the *Prelp* ^*−/−*^ BBB was most intense in the cerebellum, compared with the cortex (Figures 3E–G). This may reflect the higher levels of PRELP expression in the cerebellum. To confirm the BBB damage in *Prelp* ^*−/−*^ *,* we performed another leakage assay through injection of Texas Red conjugated 70 kDa-Dextran. Consistent with the IgG staining result, Dextran was restricted to the vasculature in wild type animals (Figures 3H–J). In comparison, we detected areas of the posterior brain where the dye was detected outside of the vasculature in *Prelp* ^*−/−*^ (Figures 3K–M).

One of the proposed mechanisms of vascular leakage is activation of EndMT of vascular endothelial cells (Sweeney et al., 2019), which is associated with a reduction in adherens and tight junctions. Recently, we demonstrated that PRELP has the ability to activate mesenchymal-to-epithelial transition (MET), resulting in the enhancement in bladder epithelial cell-cell and retinoblastoma cells (Papadaki et al., 2020; Hopkins et al., 2022). As the EndMT/MEndT mechanism is largely conserved with EMT/MET, loss of PRELP may cause vascular leakage through activation of EndMT (Saito, 2013; Hong et al., 2018).

**FIGURE 4:**
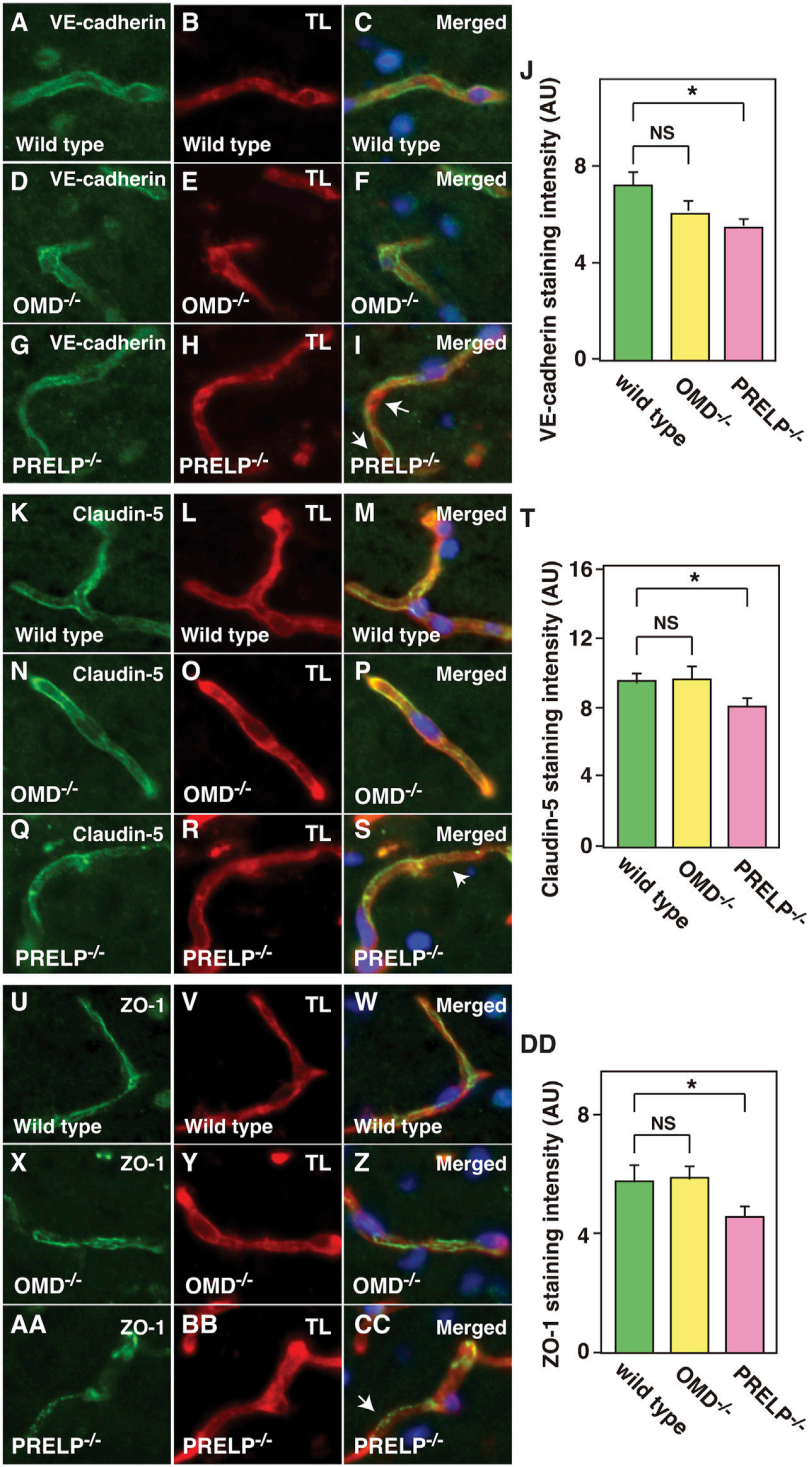
Cell-cell adhesion of cerebellum vasculature is downregulated in *Prelp* ^*−/−*^ mice. **(A–J)** Reduced VE-cadherin coverage of *Prelp* ^*−/−*^ vessels. Sections of wild-type **(A–C)**, *Omd* ^*−/−*^ **(D–F)** and *Prelp* ^*−/−*^ **(G–I)** cerebellums were stained with VE-cadherin **(A,D,G)** and TL **(B,E,H)**. Staining of *Prelp* ^*−/−*^ vessels was found to be uneven, inconsistent (arrows). Scale bar 15 μm. **(J)** VE-cadherin staining intensity was quantified (*n* = 3). (**K–T)** Weaker claudin-5 staining in *Prelp* ^−/−^ vessels. Claudin-5 and TL staining was performed in wild-type **(K–M)**, *Omd* ^*−/−*^ **(N–P)** and *Prelp* ^*−/−*^ **(Q–S)** cerebellum. Staining in *Prelp* ^−/−^ vessels was found to be more discontinuous (arrow). **(T)** Quantification of staining intensity (*n* = 3). **(U–DD)** ZO-1 staining is reduced in *Prelp* ^*−/−*^. Wild-type **(U–W)**, *Omd* ^*−/−*^ **(X–Z)** and *Prelp* ^*−/−*^ **(AA–CC)** sections were stained with ZO-1 and TL. Punctated staining along vessels were found (arrow). **(DD)** Quantification of staining intensity (*n* = 3). TL; Tomato lectin.

We previously demonstrated the cell-cell junction dysfunction in response to the OMD and PRELP expression levels in bladder cancer cells using electron microscope analysis along with immunofluorescence analysis (Papadaki et al., 2020). Therefore, we performed immunostaining against an adherens junction marker (VE-cadherin) and tight junction markers (Claudin-5 and ZO-1). In *Prelp* ^*−/−*^ *,* we observed uneven, inconsistent VE-cadherin staining in contrast to the uniform staining in wild type and *Omd* ^*−/−*^ mice (Figures 4A–I). Quantification of VE-cadherin signal revealed that there was significant reduction of VE-cadherin in *Prelp* ^*−/−*^ (Figure 4J). For claudin-5, we observed uniform membrane staining in the wild type and *Omd* ^−/−^ neurovasculature, although in *Prelp* ^*−/−*^ mice presented significantly weaker expression (Figures 4K–T). ZO-1 formed a stripe-type staining pattern around the membrane in wild type mice, which was punctuated in *Prelp* ^*−/−*^ mice (Figures 4U–CC). ZO-1 staining intensity of *Prelp* ^*−/−*^ was also significantly reduced compared to controls (Figure 4DD). These observations indicate weakened cell-cell contacts between VSMCs in *Prelp* ^*−/−*^ mice.

Next, we examined neurovascular unit (NVU) components in the *Prelp* ^*−/−*^ mouse. The basement membrane (BM) is a relatively thick layer of secreted proteoglycans, laminins, collagens and perlecan which underlies endothelial cells. These components are organised by SLRP proteins, with most SLRPs able to bind collagen (Tashima et al., 2018) and PRELP specifically shown to interact with perlecan (Bengtsson et al., 2002). Laminin staining in wildtype and *Omd* ^*−/−*^ is intense, clearly surrounding blood vessels (Supplementary Figures S1A–F). This intensity is lost in *Prelp* ^*−/−*^ mice, especially at sites with intense IgG leakage (Supplementary Figures S1G–J). Perlecan staining was also reduced in *Prelp* ^*−/−*^ (Supplementary Figures S1K–T). However, we did not observe a significant reduction of collagen IV staining in *Prekp* ^*−/−*^ compared with wild-type mice (Supplementary Figures S1U–DD), suggesting collagen IV expression was maintained independent of IgG leakage in the *Prelp* ^*−/−*^ vasculature (Supplementary Figures S1AA–CC, arrows).

Next, we examined the effect of PRELP on the distribution of pericyte and astrocyte perivascular end-feet. Aquaporin 4 (AQP4) is a water channel found on astrocyte end-feet (Haj-Yasein et al., 2011). Double staining with AQP4 and IgG in mouse cerebellum revealed that there was a significant decrease in AQP4 signal around the vasculature in *Prelp* ^*−/−*^, whereas no difference was observed in *Omd* ^−/−^ (Supplementary Figures S1EE–NN). We then examined the effect on pericytes. Pericytes were distinguished from endothelial cells by their nuclear morphology and the staining pattern of PDGFR-β. The point of association between endothelial cells with pericytes was diminished (Supplementary Figures S1OO–XX), suggesting pericyte detachment from capillaries (Supplementary Figures S1UU, arrow).

### 3.3 mRNA expression profiling and the effect of PRELP on neuroinflammation in knockout mice

**FIGURE 5:**
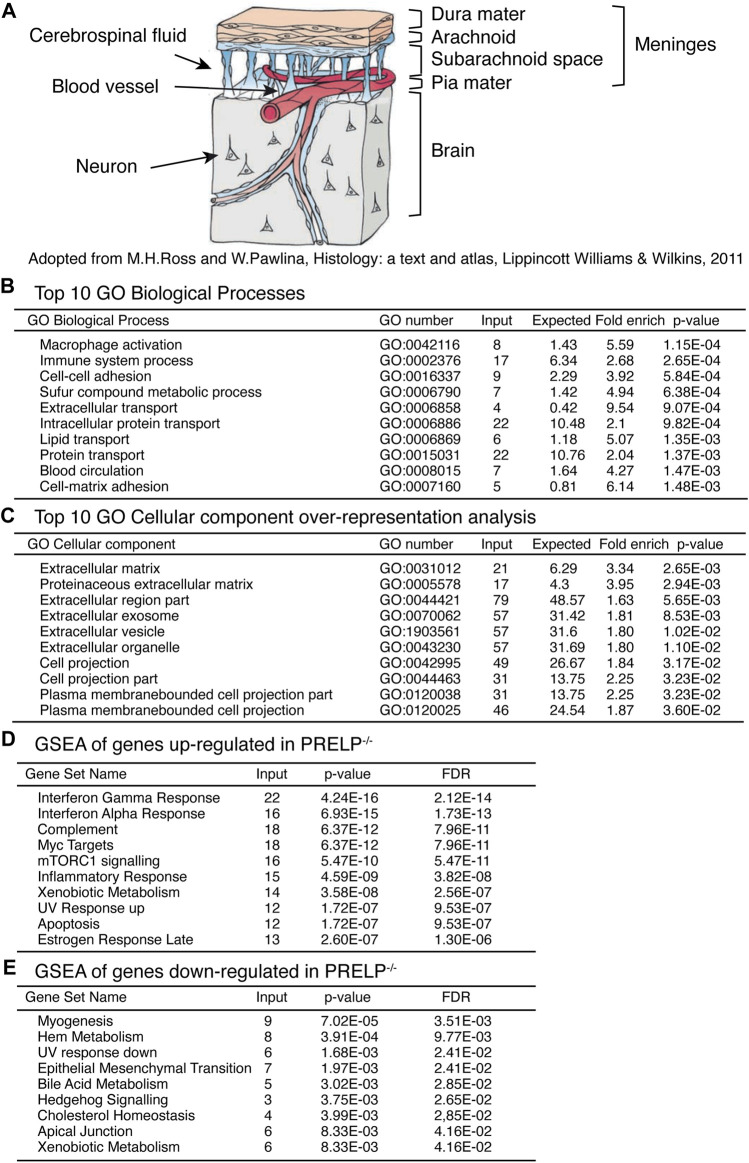
Expression profiling analysis of wild type and *Prelp* ^*−/−*^ mouse meninges. **(A)** Schematic draw of meninges. **(B)** Top 10 GO Biological Processes. **(C)** Top 10 Cellular components over-representative analysis. **(D)** GSEA of genes upregulated in *Prelp* ^*−/−*^. **(E)** GSEA of genes downregulated in *Prelp* ^*−/−*^.

The meninges contain two sites highly expressing PRELP: the meningeal vessels and cells directly contacting with cerebrospinal fluid (Figure 5A), similar to ependymal cells and choroid plexus (Figures 1L, M). To elucidate PRELP mediated biological events and their molecular mechanisms, we performed mRNA expression profiling of meninges on wild-type and *Prelp* ^*−/−*^ mice as the meninges is easily dissected with less contamination of neural tissues. However, a major disadvantage of studying the meningeal vessels is a lack of astrocytes, although as PRELP is not expressed in astrocytes surrounding neurocapillaries, this may not be important (Figures 2H–J).

RNA for expression profiling was obtained from isolated meninges from wildtype (*n* = 3) and *Prelp* ^*−/−*^ (*n* = 3) mice. We identified 288 statistically differentially expressed genes (*p* < 0.01), of which 87 genes encode extracellular proteins. Ontological analysis was performed using the tools provided by GO consortium to produce four sets of analyses; GO Biological Processes (Figure 5B), GO Cellular components (Figure 5C), Gene set enrichment analysis (GSEA) of genes upregulated in *Prelp* ^*−/−*^ (Figure 5D), and GSEA of genes downregulated in *Prelp* ^*−/−*^ (Figure 5E).

The top 10 GO Biological Processes showed that two inflammation related processes of “Macrophage activation” and “Immune system process” were the most strongly affected followed by two adhesion related process; “Cell-cell adhesion” and “Cell-matrix adhesion”. “Blood circulation” was also significantly affected. In the case of GO Cellular components, the majority of affected cellular components were related to the extracellular matrix. This is probably a reflection of the extracellular localization of PRELP. In addition, eight categories were associated with change in cellular morphology, including “Cell projection part” and “Plasma membrane bounded cell projection”. As changes to cell-cell adhesion is a major biological event that induces alterations in cell morphology, these results suggest PRELP is a regulator of cell-cell adhesion.

To further elucidate PRELP function, we utilized GSEA. We analysed upregulated and downregulated genes separately (Figures 5D, E). Interestingly, gene sets related to inflammation such as “Interferon γ response”, “Interferon α response” and “Complement” were strongly affected in *Prelp* ^*−/−*^ meninges. Cell-cell adhesion related categories, “EMT” and “Apical Junction” were significantly affected (Figure 5E), which suggests that the functional role of PRELP as regulator of partial EMT may be conserved across tissues (Papadaki et al., 2020; Hopkins et al., 2022). In summary, ontological analysis proposes two main biological roles of PRELP within the meninges: cell-cell adhesion and inflammation.

**FIGURE 6:**
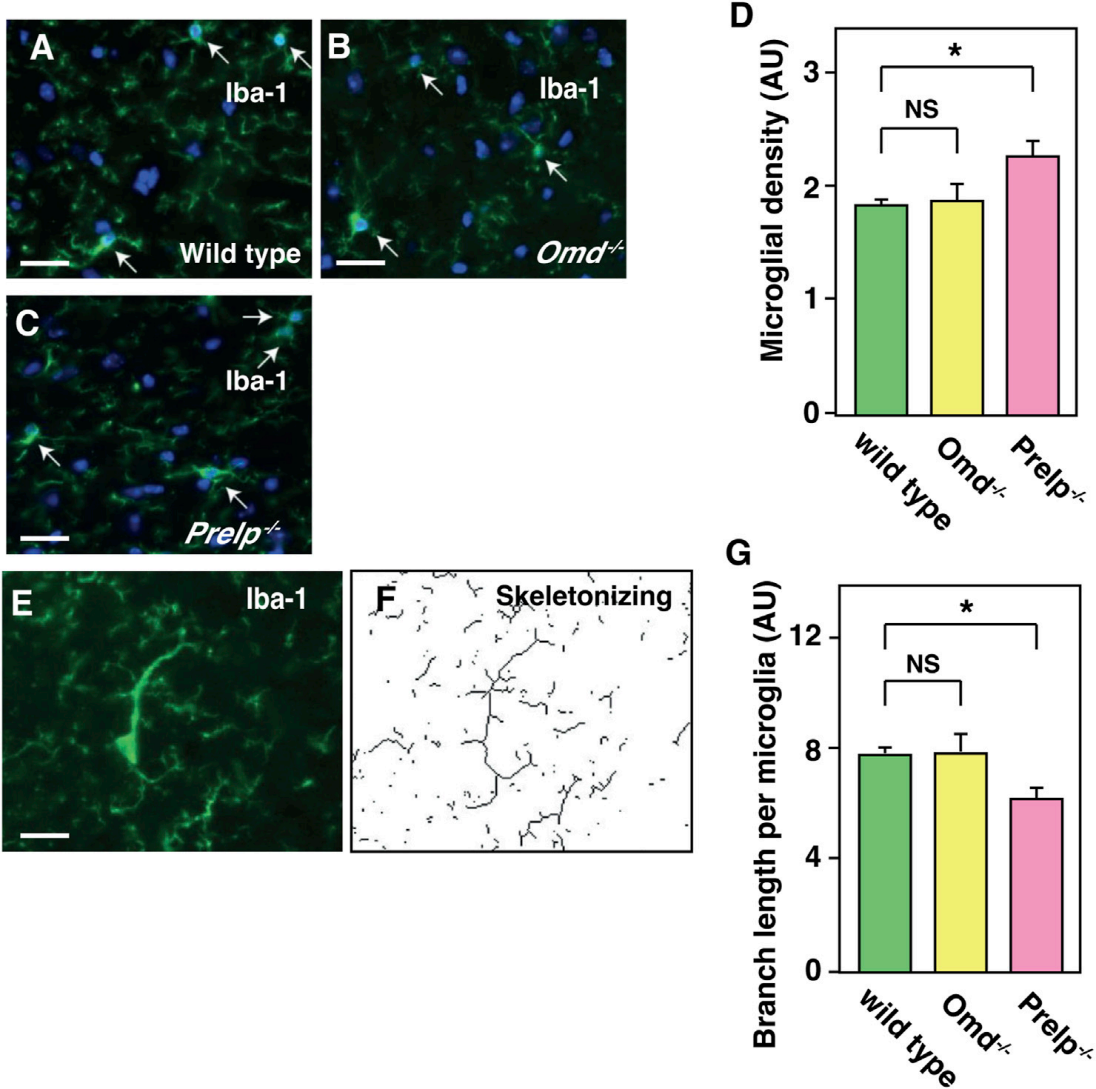
Microglia is activated in *Prelp* ^*−/−*^ mouse cerebellum. **(A–G)** Iba-1 staining of wild-type **(A)**, *Omd* ^*−/−*^ **(B)**, and *Prelp* ^*−/−*^ **(C)** cerebellum sections. Microglia were manually counted based on Iba-1 and nuclear DAPI staining (arrows). Scale bar 25 μm. **(D)** Microglial density was quantified. **(E-F)**, Skeletonizing images of Iba-1 staining to quantify microglial branch length. **(G)**, Quantification of branch length per microglial density in wild-type, *Omd* ^−/−^ and *Prelp* ^*−/−*^ brains (*n* = 3).

Expression profiling data and vascular analyses suggest that, in the *Prelp* ^*−/−*^ brain, vascular leakage may trigger inflammation. We therefore examined the status of microglia and astrocytes in the mouse cerebellum by the labelling with Iba-1 and GFAP antibodies, respectively. Firstly, the number of microglia cell bodies was counted. Irrespective of morphological change (Figures 6A–C), quantification revealed that there was an increase in the number of Iba-1 positive microglia in *Prelp* ^*−/−*^ sections (Figure 6D). Microglial functional responses in accordance with the protocol established by Morrison (Morrison and Filosa, 2013) was used with some minor modifications to examine inflammation and the sum of the branch lengths was used for quantification (Figures 6E, F). Quantification of branch length per microglial density revealed that there was a statistically significant decrease in *Prelp* ^*−/−*^ mice (Figure 6G), indicating increased microglial response and supporting the findings in our expression profiling (Figure 5B) as microglia are known as a neural type of macrophages (Masuda et al., 2020). Furthermore, we investigated the effect of PRELP on the morphology and the number of astrocytes using antibody to GFAP. However, there were no differences in astrocyte number, morphology, and staining intensity between wild-type, *Omd* ^*−/−*^, and *Prelp* ^*−/−*^ mice (Supplementary Figures S2A–E). While leakage of fluids from vasculature or ependymal layer can affect water content in the brain causing hydrocephalus (Karimy et al., 2017), we did not observe differences of water content between the wild type and *Prelp* ^*−/−*^ brain (Supplementary Figures S2F).

### 3.4 Application of PRELP protein enhances endothelial cell-cell integrity by affecting EMT-related events

To determine the role of PRELP protein in consolidating BBB integrity and elucidate its mechanism, we performed *in vitro* experiments using either PRELP conditional medium (PRELP CM) or purified recombinant PRELP protein, produced in Mimic Sf9 insect cells (Supplementary Figure S3) (Kosuge et al., 2021). All PRELP proteins showed a phenotypic effect in our assays as shown below.

**FIGURE 7:**
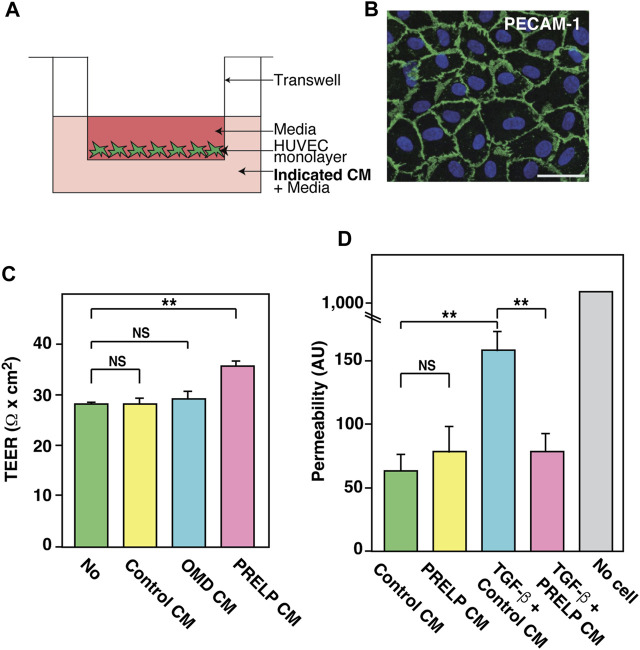
Effect of PRELP on leakage from endothelial cell monolayer. **(A,B)** Effect of PRELP on HUVEC monolayer TEER. **(A)** Schematic drawing of the assay. **(B)** Confirmation of HUVECs monolayer confluency by PECAM-1 staining. **(C)** TEER measurement. **(D)** Permeability assay was performed using HUVEC monolayer.

To ensure that PRELP is not secreted from the vasculature, we examined the expression of PRELP in HUVECs and found extremely low expression. This is consistent with RNA-seq results in other papers that showed no or very low expression in mouse brain endothelial cells (Vanlandewijck et al., 2018). Thus, we examined the effect of PRELP on a transepithelial/transendothelial electrical resistance (TEER) using a simple Human umbilical vein endothelial cells (HUVECs) monolayer (Figure 7A). After confirming HUVECs formed a monolayer via PECAM1 immunostaining (Figure 7B), we found that application of PRELP CM significantly increased TEER (Figure 7C), indicating that PRELP can enhance endothelial cell-cell integrity. Furthermore, we also examined the effect of PRELP on permeability using fluorophore-tagged 70 kDa dextran. Under these conditions, PRELP CM did not reduce permeability compared to the control but was effective at preventing TGF-β-mediated permeability, suggesting that PRELP can inhibit TGF-β signalling (Figure 7D).

EMT exists along a spectrum of different states. These partial EMT states, pEMT, are important for understanding human diseases such as cancer (Lamouille et al., 2014; Nieto et al., 2016; Aiello et al., 2018; Brabletz et al., 2018). Recently, we reported that PRELP regulates cell-cell adhesion of bladder umbrella epithelial cells and retinoblastoma cells through pEMT (Papadaki et al., 2020; Hopkins et al., 2022). Similar mechanisms, endothelial-mesenchymal transition (EndMT) has been demonstrated in vascular cells (Lamouille et al., 2014; Nieto et al., 2016; Kovacic et al., 2019). EndMT is important for regulating vascular leakage. Our expression profiling analysis of meninges showed that EMT was strongly affected by PRELP deletion (Figure 5E), suggesting that the vascular leakage in *PRELP* ^*−/−*^ mice might be caused by partial EndMT. TGF-β is a potent mediator of EMT and PRELP-mediated inhibition through the application of PRELP CM in TEER and permeability assay may increase cell adhesion via pMEndT (Figures 7C, D).

To elucidate the mechanism of PRELP action, we applied purified recombinant PRELP protein to HUVEC monolayers. HUVECs exhibit pEndMT states which contributes to cell-cell permeability (Guo et al., 2015). mRNA expression profiling was performed on HUVEC cultures incubated with PRELP for 48 h. Using Ingenuity Pathway Analysis (IPA) software, we performed ontological analysis to identify 1,903 significantly affected genes and 220 significantly affected canonical pathways. These pathways were largely classified into three categories; EndMT/cell adhesion (Supplementary Figure S4A), cancer (Supplementary Figure S4B), inflammation (Supplementary Figure S4C) and EMT related Signalling pathways. EndMT/cell adhesion events included “Regulation of the EMT pathway”, “Hepatic Fibrosis Signalling Pathway”, “Epithelial adherens junction signalling”, and “Integrin Signalling” (Supplementary Figure S4A). As EMT is strongly implicated in cancer-related pathways, we found many associated pathways such as “Molecular Mechanism of Cancer”, and “Bladder Cancer Signalling” which were also affected (Supplementary Figure S4B, (Brabletz et al., 2018). Furthermore, several interleukins related proinflammation pathways were negatively affected (IL-8, IL-3, IL-7, IL-6, and IL-4) (Supplementary Figure S4C), suggesting that PRELP may have an anti-inflammatory role as we discussed in the previous section (Effect of PRELP on neuroinflammation) and activated EMT through TGF-β, Met and Wnt signalling was observed in the “Regulation of the EMT pathway” (Supplementary Figure S5).

### 3.5 PRELP activates cell-cell adhesion of HUVEC cell culture and reverses TGF-β mediated pEndMT

The membrane localization of β-catenin, an intracellular protein directly associated with cadherin molecules, was enhanced by PRELP (Supplementary Figure S6A–F). These data indicate that PRELP enhances adherens junction formation and/or stability. We examined the effect of PRELP on tight junctions using ZO-1 (Supplementary Figures S6G, H) and claudin-5 (Supplementary Figures S6K, L) staining but could not detect tight junction formation in our conditions.

TGF-β is the strongest activator of EndMT. As observed in many other biological systems, TGF-β has complex and dual roles in vascular biology. This includes a dual role as an activator and an inhibitor of BBB function in context dependent manners (Li et al., 2011; Diniz et al., 2019). Application of TGF-β to HUVECs has been reported to cause damage to endothelial cell-cell adhesion through activation of pEndMT (Guo et al., 2015). Using this system, we examined the effect of PRELP on TGF-β mediated pEndMT. As shown in Supplementary Figure S6 , 20 ng/mL TGF-β resulted in the increase of β-catenin membrane staining (Supplementary Figure S6C). PRELP application reversed all TGF-β mediated effects (Supplementary Figures S6D, J, N) suggesting that PRELP can rescue TGF-β mediated vascular damage in association with inhibition of pEndMT and all of these may be associated with activation of pEndMT (Guo et al., 2015).

## 4 Discussion

### 4.1 PRELP is a novel regulator of pEndMT in vascular homeostasis

Our *in vitro* studies show that PRELP activates EndMT and enhances cell-cell adhesion of endothelial cells which may occur in a TGF-β-dependent manner. Conversely, the *in vivo* phenotype in *Prelp* ^*−/−*^ mice also demonstrated pEndMT activation and reduced cell-cell adhesion in the cerebellum. Furthermore, involvement of PRELP mediated regulation of EndMT in both *in vivo* and *in vitro* was confirmed by expression profiling of PRELP-treated HUVECs and *Prelp* ^*−/−*^ meninges. As we previously mentioned in the result section in Figure 2A, the analysis of *PRELP* expression in pericytes and vSMCs, but not in endothelial cells has been confirmed by published single cell mRNA expression profiling data (Zeisel et al., 2015; He et al., 2016; Vanlandewijck et al., 2018). Our RNA-seq result shows that there are very low expression levels of PRELP in HUVECs. One paper demonstrated that the proteoglycan agrin, which is widely expressed in neurons and microvascular basal lamina in the rodent and avian central nervous system (Donahue et al., 1999) regulated the junction proteins of VE-cadherin, β-catenin, and ZO-1, and stabilized junctional localization of VE-cadherin *in vivo* (Steiner et al., 2014). This indicates that proteoglycans, including PRELP can maintain BBB function by regulating and stabilizing junction protein expression without being express in endothelial cells.

Recently we showed that PRELP activates bladder epithelial cell-cell adhesion by activation of MET. This was mediated via direct inhibition of TGF-β and/or EGF mediated pEMT (Papadaki et al., 2020).

Indeed, an independent study demonstrated that PRELP can antagonize TFG-β (Chacon-Solano et al., 2022). This activity is important for maintenance of the blood-urine barrier (Kreft et al., 2010). In addition to the BBB, the blood-CSF barrier, where cell-cell adhesions between choroid plexus and ventricle ependymal cells, plays an important role in separating the brain from non-brain tissues (Liddelow, 2015). These observations indicate that PRELP may have a conserved function to maintain biological barriers by regulating either pEndMT or pEMT.

### 4.2 The mechanism of PRELP deletion mediated leakage from BBB

In the *Prelp* ^*−/−*^ mouse brain, NVU components, BM proteins, pericytes and astrocyte endo-feet, were downregulated. Downregulation of BBB components has been frequently reported to cause leakage of the BBB. For example, mice lacking laminin α2 or laminin γ1 display significant abnormalities to brain vasculature integrity (Menezes et al., 2014; Yao et al., 2014; Gautam et al., 2016). Ablation of PDGF-β results in reduction of pericyte coverage and subsequent decreased vascular density and increased vascular permeability and vessel diameter (Bjarnegard et al., 2004). Interestingly, deletion of CD146, an EMT inducer in pericytes, results in reduced coverage of pericytes around vasculature (Zeng et al., 2012; Chen et al., 2017), suggesting that EMT/EndMT might be involved in interaction between pericytes and endothelial cells. We observed a decrease in the intensity of AQP4 staining in *Prelp* ^*−/−*^ *,* which is often found in other BBB breakdown model mice (Menezes et al., 2014; Gautam et al., 2016).

These observations suggest that in addition to the direct effect of PRELP-mediated regulation of cell-cell adhesion between endothelial cells, PRELP may also indirectly control BBB integrity through regulation of the NVU components.

### 4.3 The mechanism of PRELP deletion mediated neuroinflammation

Our expression profiling analysis of meninges and immunohistochemical analysis of microglia indicated the presence of neuroinflammation in the *Prelp* ^*−/−*^ brain. This is likely to be an indirect effect, since blood proteins leaking into the brain tissue cause neuroinflammation and can perpetuate to neurodegenerative disorders (Weiss et al., 2009; Sweeney et al., 2019). Indeed, our expression profiling of HUVECs demonstrated that PRELP application inhibited proinflammatory interleukins including IL-8, IL-3, IL-7, IL-6, and IL-4. Moreover, PRELP has previously been reported to bind to C9 complement to prevent the formation of the membrane attack complex (Happonen et al., 2012) and acts as a potent inhibitor of complement-mediated damage in mouse eyes (Birke et al., 2014). Indeed, “Complement” pathway was also significantly affected in *Prelp* ^*−/−*^ meninges. Furthermore, the importance of EMT/EndMT in inflammation has been recognized (Lopez-Novoa and Nieto, 2009; Cho et al., 2018). Together these observations suggest that PRELP may regulate to neural inflammation as an anti-inflammatory factor.

Severe neural inflammation can lead to alterations in the BBB (Gaillard et al., 2003). However, our results provide evidence that inflammation in *Prelp* ^*−/−*^ mice was relatively mild and may not be sufficient to cause the BBB damage observed. We did not observe activation of astrocytes in *Prelp* ^*−/−*^ brain, common inflammation markers or abnormal behavior of mice and there was no change in water content (unpublished data). A cumulation of factors, including PRELP, may therefore be required to generate damaging levels of neuroinflammation.

In conclusion, our results indicate that PRELP, a secreted novel regulator of pEndMT, enhances BBB integrity, maintains vasculature homeostasis in the brain and might be a potential treatment for neural diseases associated with BBB leakage and neuroinflammation.

## 5 Limitation of the study

There are some limitations in analysing the effect of glycosylated proteins. First, proteoglycans including PRELP have different formulas based on varied amounts of post-translational sugar chain modifications which can modifications vary among species. Although two sources of PRELP proteins showed almost identical phenotypes, sugar modification may affect activity.

## Data Availability

The datasets presented in this study can be found in online repositories. The names of the repository/repositories and accession number(s) can be found below: https://www.ncbi.nlm.nih.gov/geo/, GSE199122.
