# Supplementary figures and images for: PRELP secreted from mural cells protects the function of blood brain barrier through regulation of endothelial cell-cell integrity
Source: Front Cell Dev Biol. 2023 Oct 23;11:1147625. doi: 10.3389/fcell.2023.1147625 (PMC10626469; doi:10.3389/fcell.2023.1147625)

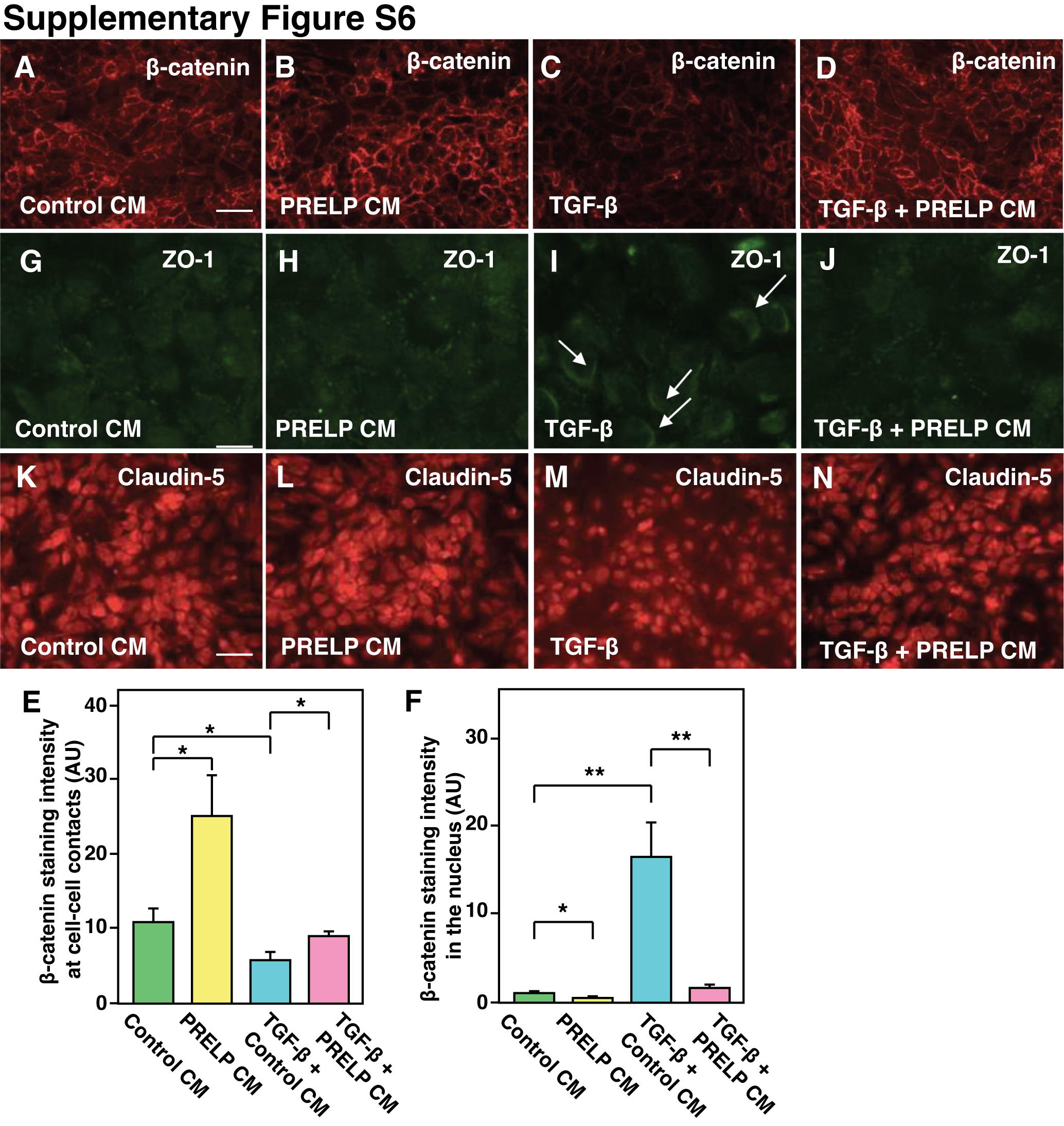

Supplement: Supplementary file 1 [file Image6.TIF]

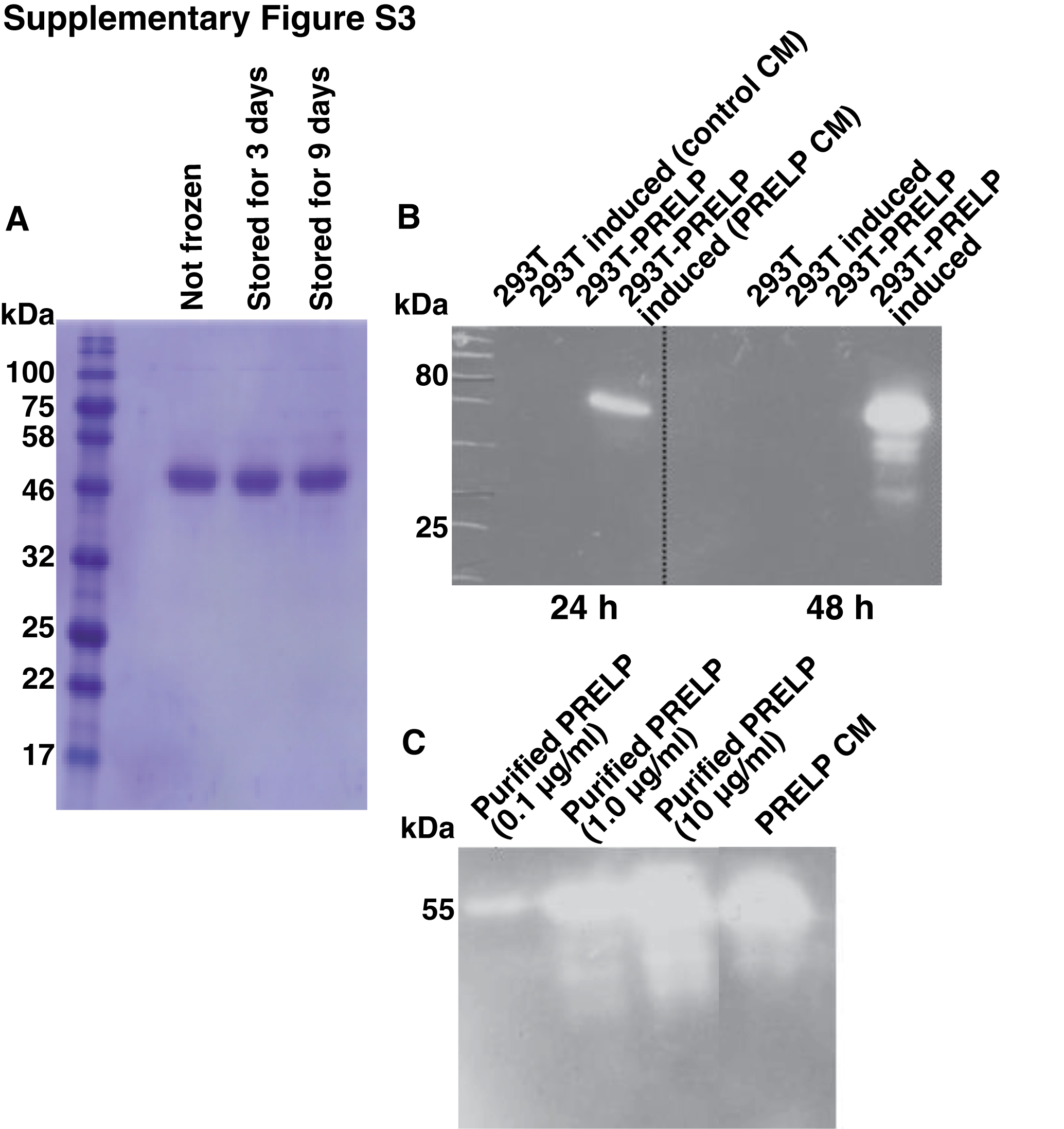

Supplement: Supplementary file 3 [file Image3.TIF]

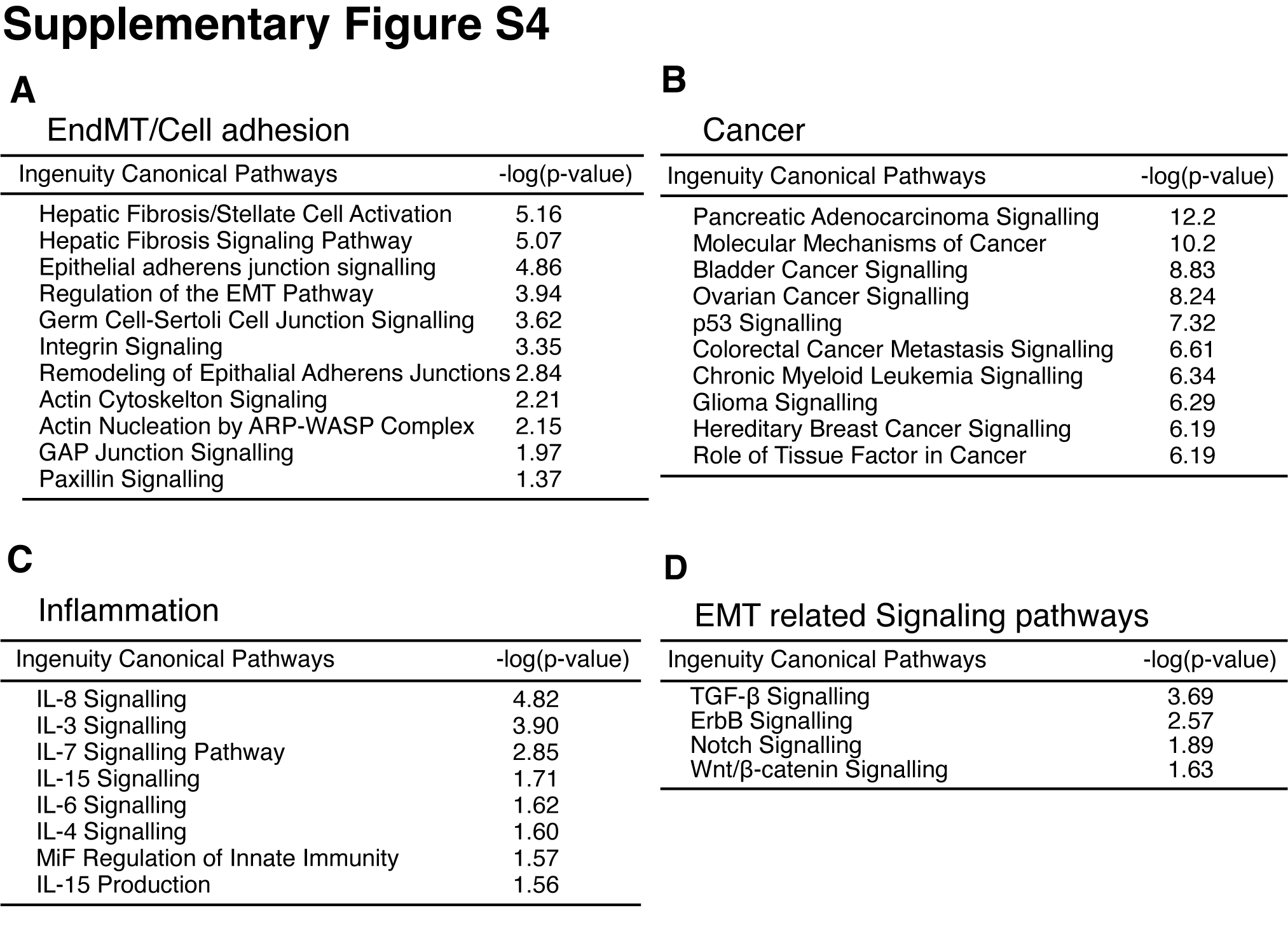

Supplement: Supplementary file 4 [file Image4.TIF]

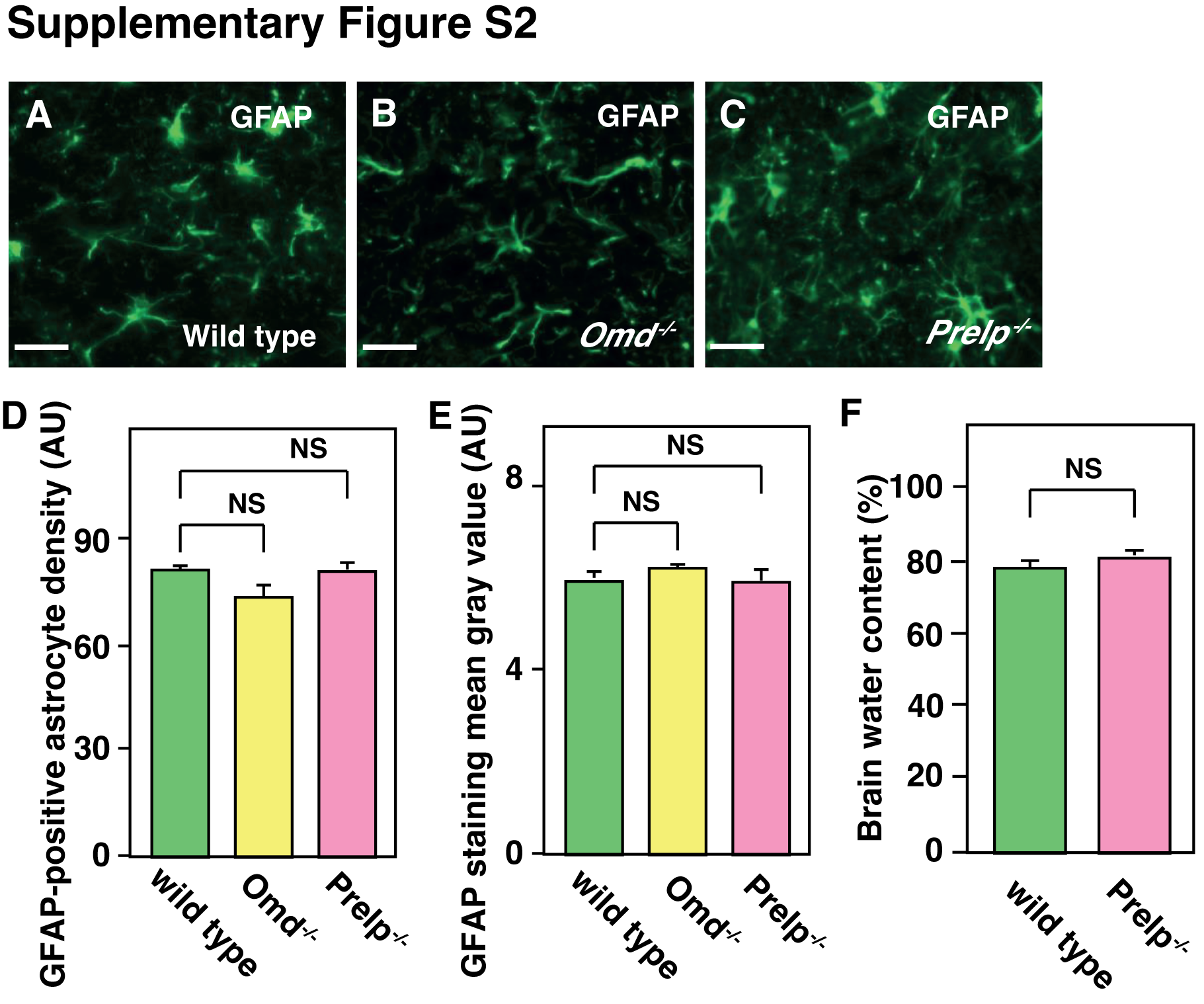

Supplement: Supplementary file 5 [file Image2.TIF]

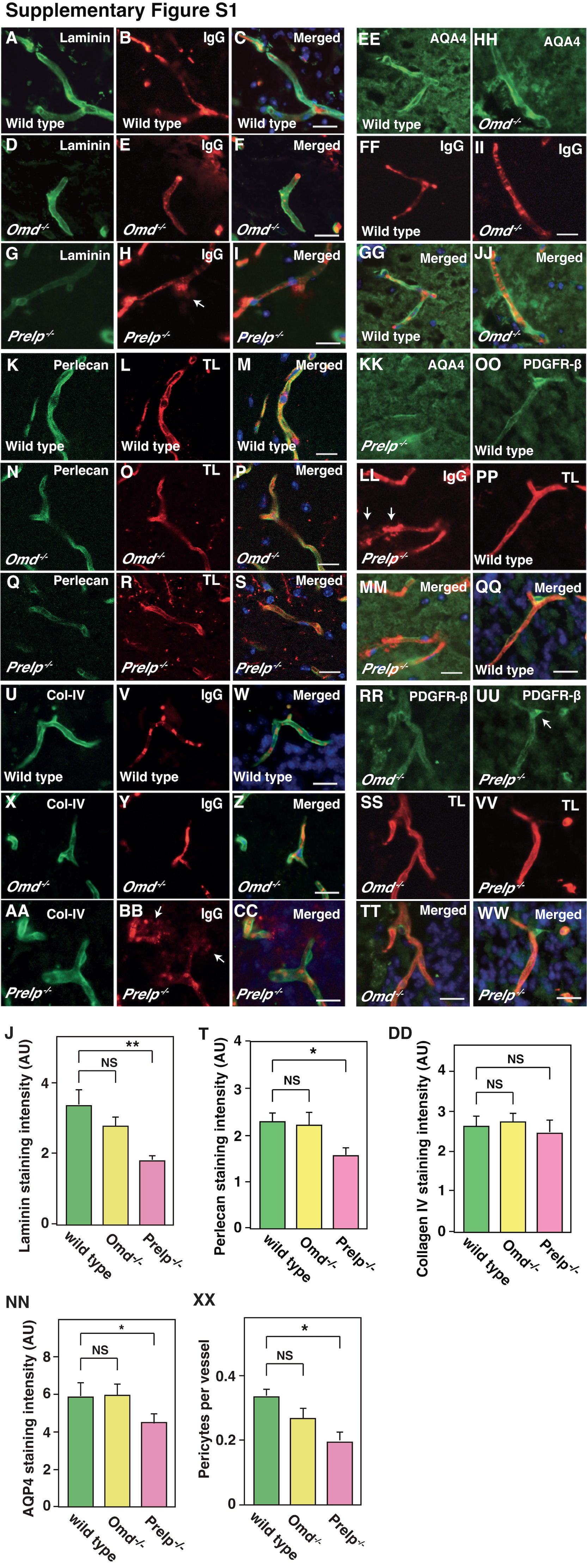

Supplement: Supplementary file 6 [file Image1.TIF]

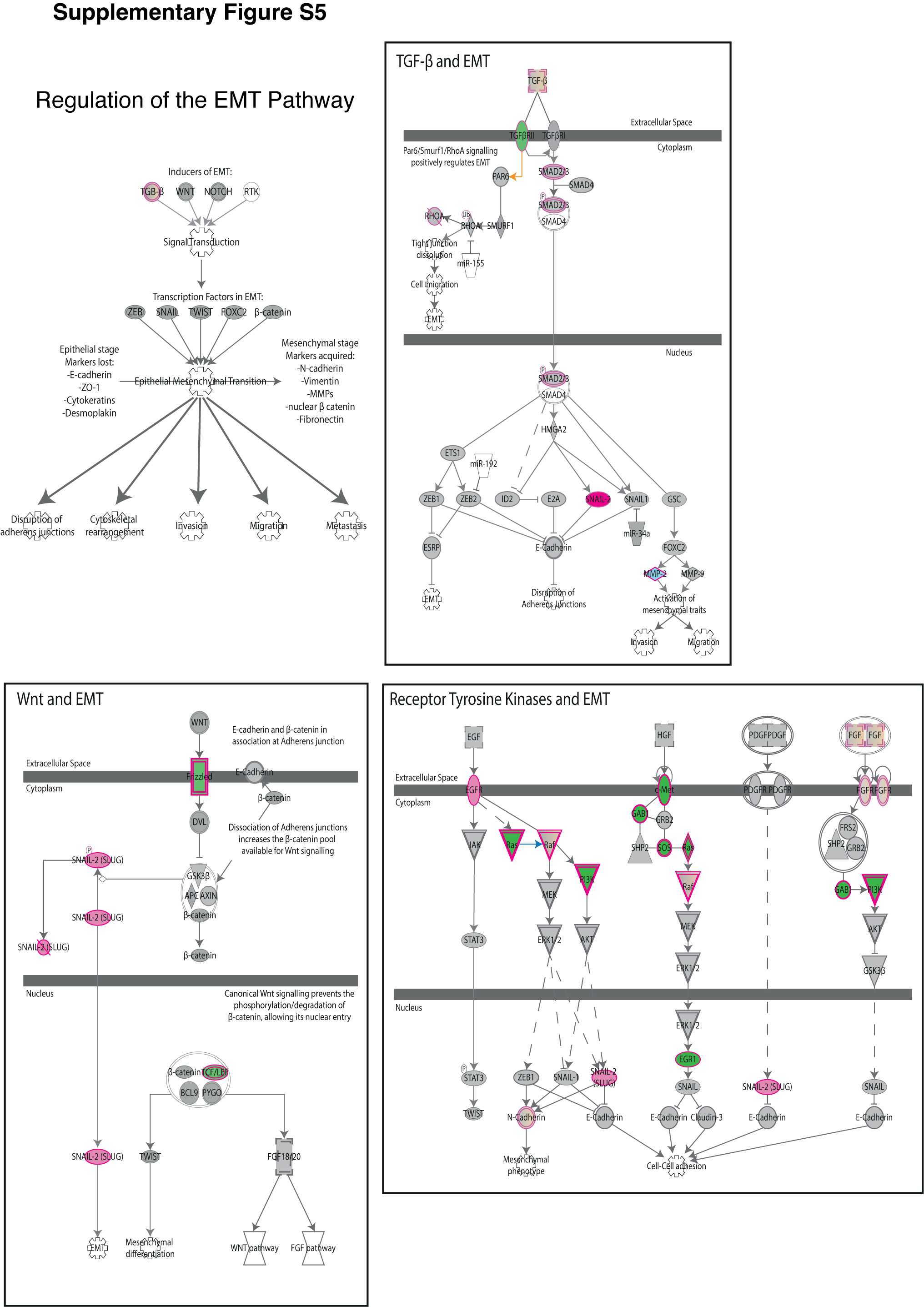

Supplement: Supplementary file 9 [file Image5.TIF]
