# Supplementary material for: PRELP secreted from mural cells protects the function of blood brain barrier through regulation of endothelial cell-cell integrity
Source: Front Cell Dev Biol. 2023 Oct 23;11:1147625. doi: 10.3389/fcell.2023.1147625 (PMC10626469; doi:10.3389/fcell.2023.1147625)
Supplement: Supplementary file 2 [file Table1.DOCX]

**Supplementary Table S1. List of antibodies, reagents, data accession number, and software used in this study**

| Antibodies | | |
| --- | --- | --- |
| Rabbit anti-PRELP antibody | This paper | #15 |
| Rabbit anti-VE-cadherin antibody | Abcam | #ab33168 |
| Rabbit anti-Claudin-5 antibody | Abcam | #ab15106 |
| Rabbit anti-ZO-1 antibody | Invitrogen | #402300 |
| Rabbit anti-β-actin antibody | CST | #3700 |
| Rabbit anti-Paxillin antibody | Abcam | #ab32084 |
| Rabbit anti-sm22-a antibody | ProteinTech | #104931 |
| Rabbit anti-PECAM1 antibody | Abcam | #ab28364 |
| Rabbit anti-a-SMA antibody | Abcam | #Ab5694 |
| Mouse anti-b-Galactosidase antibody | Promega | #Z3781 |
| Rabbit anti-GFAP antibody | Sigma | #G4546 |
| Rabbit anti-NG2 antibody | Millipore | #AB5320 |
| Anti-Isolectin IB4 antibody | Thermo Fisher | #I12411 |
| Rabbit anti-Laminin antibody | Abcam | #ab11575 |
| Rabbit anti-Perlecan antibody | Abcam | #Ab2501 |
| Rabbit anti-Collagen IV antibody | Abcam | #Ab6586 |
| Rabbit anti-Aquaporin 5 antibody | Abcam | #ab104751 |
| Rabbit anti-PDGFR-b antibody | CST | #3169 |
| Rabbit anti-Iba-1 antibody | Abcam | #ab178680 |
| Rabbit anti-b-catenin antibody | CST | #8480 |
| Mouse anti-IgG | CST | #7076 |
| Chemicals, Peptides, and Recombinant Proteins | | |
| X-gal | Invitrogen | K146501 |
| 70 kDa Dextran-Texas Red | Thermo Fisher | D1864 |
| TGF-b | R&D Systems | 240-B-002 |
| Rhodamine Phalloidin | Thermo Fisher | #R415 |
| Critical Commercial Assays | | |
| ARCTURUS PicoPure RNA Isolation kit | Thermo Fisher | KIT0204 |
| PureLinkTM RNA Mini Kit | Thermo Fisher | #12183018A |
| Taq DNA Polymerase, native w/W1 | Invitrogen | #18038018 |
| QIAGEN Multiplex PCR Kit | QIAGEN | #206143 |
| *In vitro* vascular permeability assay kit | Merck | ECM642 |
| Deposited Data | | |
| Next generation sequencing | This paper | GSE199122 |
| Experimental Models: Organisms/Strains | | |
| Mouse: C57BL/6J | Takeda Pharmaceutical Company, Ltd. | N/A |
| Software and Algorithms |  |  |
| ImageJ | NIH software | https://imagej.net |
| STATA | Version 8.0; StataCorp, College Station, TX, USA | https://www.stata.com/stata8/ |
| Galaxy | Version 20.01 | https://usegalaxy.org/ |
| Illumina’s bcl2fastq Conversion Software | Illumina | https://emea.support.illumina.com/sequencing/sequencing_software/bcl2fastq-conversion-software.html?langsel=/gb/ |
| RNA-STAR | Version 2.5.2b | GitHub: https://github.com/alexdobin/STAR |
| Je-suite | Version 1.2.1 | https://gbcs.embl.de/portal/tiki-index.php?page=Je |
| Qiagen’s Ingenuity Pathway Analysis IPA | Qiagen, ver. 48207413 | N/A |
| AngioTool | https://doi.org/10.1371/journal.pone.0027385 | N/A |
