# Supplementary material for: PRELP secreted from mural cells protects the function of blood brain barrier through regulation of endothelial cell-cell integrity
Source: Front Cell Dev Biol. 2023 Oct 23;11:1147625. doi: 10.3389/fcell.2023.1147625 (PMC10626469; doi:10.3389/fcell.2023.1147625)
Supplement: Supplementary file 7 [file Table2.DOCX]

**Supplementary Table S2. List of primers used in this study**

| Oligonucleotides | | |
| --- | --- | --- |
| Genotyping primer OMD-A: GGGAATGCTTTGACTTTCTGAGTTA | This paper | N/A |
| Genotyping primer OMD-B2: TGAAGCATTGATGCCTGCTA | This paper | N/A |
| Genotyping primer LacZ_5756F  CGGTCGCTACCATTACCAGT | This paper | N/A |
| Genotyping primer PRELP-A: CACTGCAGGAAGAGTCATCTTTTCT | This paper | N/A |
| Genotyping primer PRELP-B: TACACTTTCTCCCAGCTTCTATTCC | This paper | N/A |
| Genotyping primer PRELP-C:  GTTGAGCAGATTTTGGATGTCACT | This paper | N/A |
| Genotyping primer LacZ-B: GGATAGGTCACGTTGGTGTAGATG | This paper | N/A |
