# Supplementary material for: PRELP secreted from mural cells protects the function of blood brain barrier through regulation of endothelial cell-cell integrity
Source: Front Cell Dev Biol. 2023 Oct 23;11:1147625. doi: 10.3389/fcell.2023.1147625 (PMC10626469; doi:10.3389/fcell.2023.1147625)
Supplement: Supplementary file 8 [file Table3.DOCX]

Supplementary Material

PRELP secreted from mural cells protects the function of blood brain barrier through regulation of endothelial cell-cell integrity

Hongorzul Davaapil^1,7,8^, Ken Asada^2,3,7^, Jack Hopkins^1,7^, Nadia Bonnin^1^, Vasiliki Papadaki^1,8^, Alex Leung^1^, Hirofumi Kosuge^4^, Takumi Tashima^4,8^, Makoto Nakakido^4^, Ryohei Sekido^1^, Kouhei Tsumoto^4^, Ryuji Hamamoto^2,3^, Mandeep S. Sagoo^1,5,6^, Shin-ichi Ohnuma^1*^

*** Correspondence:** Shin-ichi Ohnuma: s.ohnuma@ucl.ac.uk

## Supplementary Figures

**
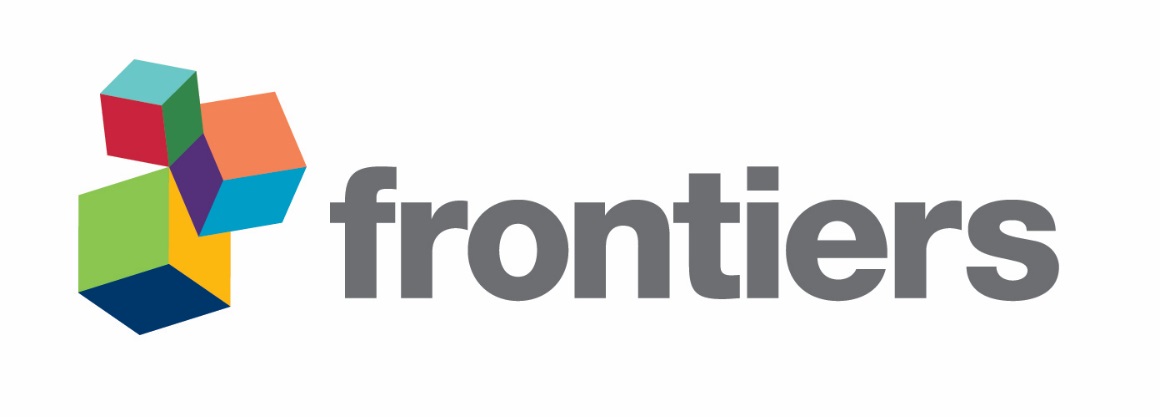
**

**Figure legends**

**Supplementary Figure S1.** BBB components are damaged in *Prelp^-/-^* mouse cerebellum. **(A-J)** Reduction of laminin in *Prelp*^-/-^ mouse. Wild-type **(A-C)**, *Omd-/-* **(D-F)** and *Prelp-/-* **(G-I)** cerebellum were stained for laminin **(A,D,G)** and IgG for leakages **(B,E,H)**. (**C,F,I)** Merged image. (**J)** Quantification of Laminin staining (*n* = 3). Areas of IgG leakage are indicated by arrows. Scale bar: 20 μm. (**K-S)** Cerebellum from Wild-type **(K-M)**, *Omd-/-* **(N-P)** and *Prelp-/-* **(Q-S)** were stained with perlecan antibody **(K,N,Q)** and TL **(L,O,R)**. (**M,P,S)** Merged image. **T,** Quantification of Perican staining (*n* = 3). Cerebellum from Wild-type **(U-W)**, *Omd-/-* **(X-Z)** and *Prelp-/-* **(AA-CC)** were stained with collagen IV antibody **(U,X,AA)** and IgG antibody **(V,Y,BB)**. Scale var: 20 μm. (**W,Z,CC)** Merged, image. (**DD)** Quantification of collagen IV staining (*n* = 3). Areas of IgG leakage are indicated by arrows. Scale bar: var: 20 μm. (**EE-MM)** double-Immunostaining. Double staining of AQA4 **(EE, HH, KK)** and IgG **(FF,II,LL)** in wild-type **(EE-GG)**, *Omd-/-* **(HH-JJ)** and *Prelp-/-* **(KK-MM)** cerebellum were performed. **(GG,JJ,MM)** Merged image. Arrows show areas of IgG leakage. (**NN)** Quantification of aquaporin-4 staining (*n* = 3). Scale bar: 20 μm. Quantification reveals that there is reduced aquaporin-4 staining around blood vessels in *Prelp*-/-. Scale bar: 20 μm. (**OO-XX)** PDGFR-β **(OO,RR,UU)** and TL **(PP,SS,VV)** double stain allows us to visualize pericytes and blood vessels in wild-type **(OO-QQ)**, *Omd-/-* **(RR-TT)** and *Prelp-/-* **(UU-WW)**. **QQ,TT,WW,** Merged. Pericytes detaching from blood vessel can be observed (arrow). (**XX)** Quantification of pericytes per vessel (*n* = 3). Scale bar: 20 μm.

**Supplementary Figure S2.**

(**A-F)** Wild-type **(A)**, *Omd-/-* **(B)** and *Prelp-/-* **(C)** sections were stained with GFAP. Scale bar 25 μm**. (D,E)** Quantification. Astrocyte count **(D)** and mean grey value **(E)** were quantified. (**F)** Brain water contents of wild type and *Prelp^-/-^* mice were determined.

**Supplementary Figure S3.** PRELP proteins used in this paper. **(A)** CBB staining of purified recombinant PRELP (prPRELP) expressed in Mimic Sf9 insect cells. The prPRELP is stable for frozen storage. (**B)** Western blotting of control CM and PRELP CM. There is no detectable PRELP protein in control CM. Doxycycline induced significant amount of PRELP protein expression. We used PRELP CM after 24 h induction because there is degraded protein at 48 h. (**C)** Quantification of PRELP protein in PRELP CM. 1.2 μg/ml PRELP protein was included in PRELP CM.

**Supplementary Figure S4.** Expression profiling of PRELP application on HUVECs. **(A-D)** The effect of purified recombinant PRELP protein on HUVEC monolayer culture was examined by mRNA expression profiling. After ontological analysis using Ingenuity software, significantly affected Ingenuity Canonical pathways were classified into four categories: EndMT/Cell adhesion **(A)**, Cancer **(B)**, Inflammation **(C)**, and EMT related Signalling pathways **(D)**.

**Supplementary Figure S5.** Mechanism of Epithelial-mesenchymal transition. Schematic drawing of “Regulation of the EMT pathway” Ingenuity Canonical Pathway of expression profiling data obtained from purified PRELP application vs control in HUVEC monolayer culture. The image was created by Ingenuity Pathway Analysis according to their rule.

**Supplementary Figure S6.** Effect of PRELP on cell-cell adhesion of HUVEC monolayer. (**A-F)** Control CM **(A)**, PRELP CM **(B)**, TGF-β **(C)**, or TGF-β + PRELP CM **(D)** was applied to HUVECs and β-catenin antibody staining was performed. Scale bar: 30 μm. (**E)** β-catenin staining intensity at cell-cell contacts was determined. (**F)** β-catenin staining intensity in the nuclei was determined. (**G-J)** Control CM **(G)**, PRELP CM **(H)**, TGF-β **(I)**, or TGF-β + PRELP CM **(J)** was applied to HUVECs and ZO-1 antibody staining was performed. Arrows indicate segregation of ZO-1 from cell-cell contacts. Scale bar: 25 μm. (**K-N)** Control CM **(K)**, PRELP CM **(L)**, TGF-β **(M)**, or TGF-β + PRELP CM **(N)** was applied to HUVECs and claudin-5 antibody staining was performed.
